# Supplementary figures and images for: Inhibition of Toll-like Receptors Alters Macrophage Cholesterol Efflux and Foam Cell Formation
Source: Int J Mol Sci. 2024 Jun 20;25(12):6808. doi: 10.3390/ijms25126808 (PMC11203583; doi:10.3390/ijms25126808)

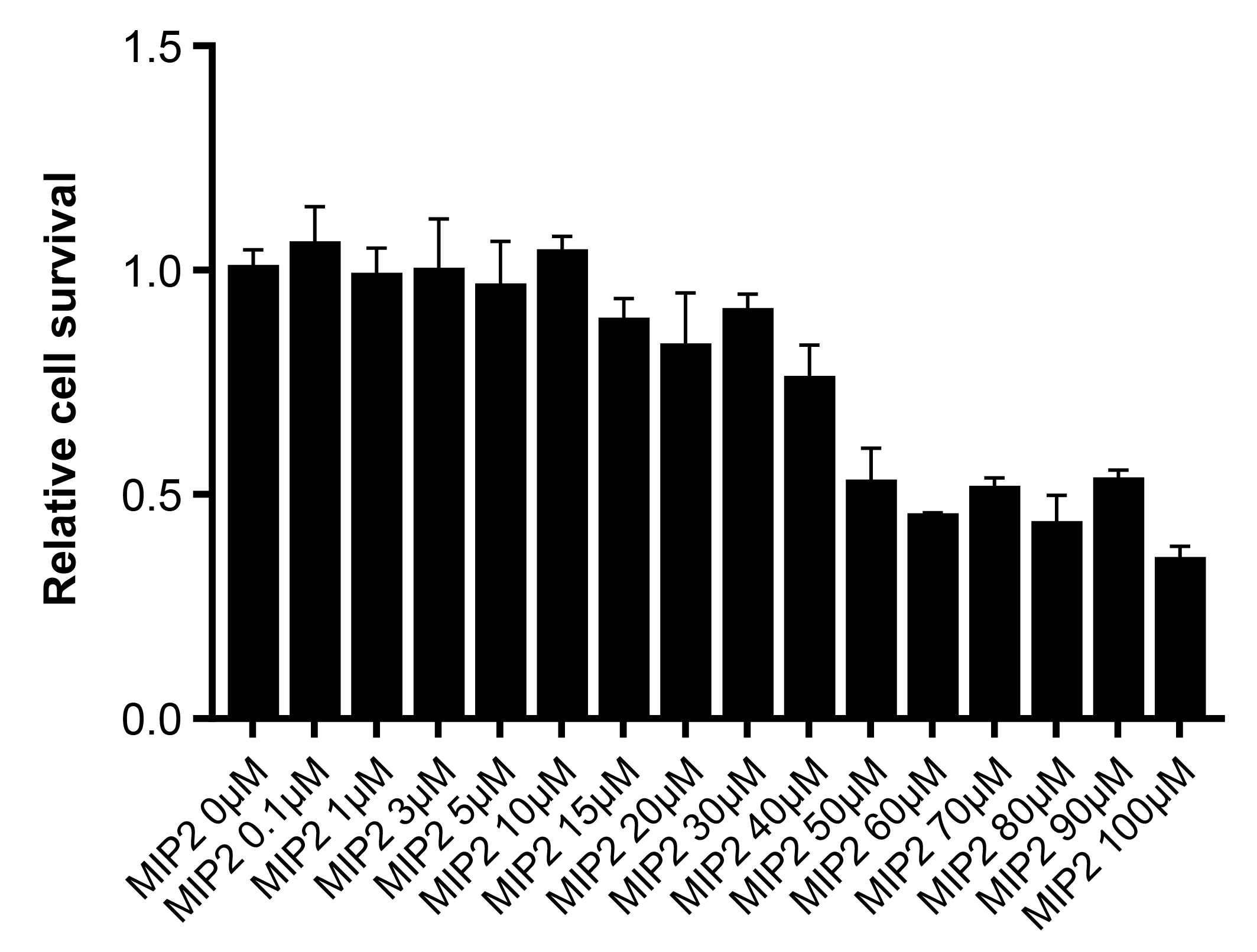

Supplement: Supplementary file 1 [file ijms-25-06808-s001.zip › ijms-3048365-supplementary.tif]
